# An INS and UWB Fusion-Based Gyroscope Drift Correction Approach for Indoor Pedestrian Tracking

**DOI:** 10.3390/s20164476

**Published:** 2020-08-10

**Authors:** Qinglin Tian, Kevin I-Kai Wang, Zoran Salcic

**Affiliations:** Department of Electrical, Computer, and Software Engineering, The University of Auckland, Auckland 1010, New Zealand; kevin.wang@auckland.ac.nz (K.I.-K.W.); z.salcic@auckland.ac.nz (Z.S.)

**Keywords:** inertial navigation system, ultra-wideband, information fusion, drift correction, pedestrian tracking

## Abstract

Information fusion combining inertial navigation and radio frequency (RF) technologies, is commonly applied in indoor positioning systems (IPSs) to obtain more accurate tracking results. The performance of the inertial navigation system (INS) subsystem is affected by sensor drift over time and the RF-based subsystem aims to correct the position estimate using a fusion filter. However, the inherent sensor drift is usually not corrected during fusion, which leads to increasingly erroneous estimates over a short period of time. Among the inertial sensor drifts, gyroscope drift has the most significant impact in determining the correct orientation and accurate tracking. A gyroscope drift correction approach is proposed in this study and is incorporated in an INS and ultra-wideband (UWB) fusion IPS where only distance measurements from UWB subsystem are used. The drift correction approach is based on turn detection to account for the fact that gyroscope drift is accumulated during a turn. Practical pedestrian tracking experiments are conducted to demonstrate the accuracy of the drift correction approach. With the gyroscope drift corrected, the fusion IPS is able to provide more accurate tracking performance and achieve up to 64.52% mean position error reduction when compared to the INS only tracking result.

## 1. Introduction

Accurate and reliable indoor positioning and tracking is a key enabler for a number of location-based services including navigation, elderly healthcare, emergency responder, etc. [1]. The performance of global positioning systems deteriorates severely in indoor environment and urban canyon due to signal attenuation [2]. The underlying principle of trilateration inspired the implementation of indoor positioning systems (IPSs) utilizing different wireless technologies including Wi-Fi, Bluetooth, radio frequency identification (RFID) and ultra-wideband (UWB). With wireless anchor nodes installed at known indoor positions, the distance between a mobile node with unknown position and each anchor node is calculated based on the wireless signal propagation time, i.e., time-of-flight (ToF). The position of the mobile node is then solved by combining the distance measurements and anchor locations by trilateration [3]. Despite the achieved positioning and tracking performance, the major drawback of wireless technology-based IPS is the requirement of installed infrastructure in the indoor environment. The number of required anchor nodes varies depending on the coverage of the IPS and the required accuracy [4,5,6].

Self-contained inertial navigation system (INS) [7] is an alternative solution utilizing an inertial measurement unit (IMU) for position tracking with the advantage that no infrastructure is required. The IMU typically consists of components including accelerometer, gyroscope and magnetometer. Starting from a known location, INS keeps track of the location of the IMU by continuously accumulating the displacement. The traveled distance is calculated using accelerometer data and the moving direction is obtained from magnetometer or gyroscope. Magnetometer is vulnerable to unknown magnetic disturbance which may very likely exist in indoor environments, whereas gyroscope is less influenced by environmental uncertainties. Smartphones are gaining popularity in recent years as an ideal platform to develop self-contained IPS for pedestrians [7,8,9] with the advances of microelectro–mechanical systems. Typically, a developed IPS combining accelerometer and gyroscope is able to provide accurate location tracking up to 100 m and the accuracy deteriorates significantly over time afterwards mainly due to sensor drift [10]. The drift in accelerometer data may lead to more than 10 m error within one minute when estimating traveled distance using double integration without correction [11]. Step-based pedestrian dead reckoning (PDR) is less influenced by accelerometer drift, but the performance is heavily affected by gyroscope drift. Although tactical grade gyroscope is able to keep a low drift over time at 1.8° per hour [12], the consumer grade gyroscope in off-the-shelf devices will accumulate drift quickly and may have more than 10° drift after 90 s [10].

In order to enhance the performance of INS-based IPS, fusion algorithms employing Kalman filter (KF), extended Kalman filter (EKF) and particle filter (PF) are proposed [13] to complement INS with wireless technologies. The UWB technology is extensively studied recently since it is able to provide precise distance measurement between two UWB-enabled nodes with error in the order of centimeters for distances up to 100 m in ideal cases [14]. The distance is calculated based on ToF by implementing a two-way ranging algorithm [15]. A number of works design INS and UWB fusion IPSs (FIPSs) for more reliable tracking performance [16,17,18].

There are two main issues observed in most FIPSs. First, the INS and UWB FIPS requires that the UWB subsystem is able to provide a position estimation independently, which inevitably requires at least four UWB anchor nodes to obtain a 3D position. Given the fact that UWB nodes are much more expensive than other wireless nodes/devices, the deployment cost for a large-scale INS and UWB FIPS will be prohibitively high. Second, the gyroscope drift is not corrected in the INS subsystem. When the gyroscope drift becomes significant over a long tracking period, the overall tracking performance of the FIPS will also deteriorate significantly.

The motivation of the paper is to address the two issues with a cost-effective approach to enable large-scale deployment of INS and UWB FIPS. It is observed that the heading orientation obtained from gyroscope is stable when the pedestrian is walking in a straight line and the drift accumulates when a turn is made. In this paper, a turn detection-based gyroscope drift correction approach is proposed and incorporated with an INS and UWB FIPS, where only distance measurements from UWB subsystem are used [19]. The contributions of the paper are listed as follows:

1. A novel gyroscope drift estimation algorithm is proposed by only utilizing heading orientations and location estimations from sensor fusion system, which can be readily applied to and integrated with other systems using different sensing hardware and algorithms (further discussed in Section 2);
2. The number of required UWB anchors in the fusion system can be significantly reduced by fusing only distance measurements from arbitrary number of available anchors. Conventional use of UWB in other published approaches requires that every possible location in the deployment area is covered by at least four UWB anchors. The total number of UWB anchors subsequently increases significantly for larger deployment area. When only fusing distance measurements, large area can be easily covered by significantly fewer anchors.
3. Practical pedestrian tracking is designed and conducted to verify the proposed approach. The results demonstrate that the FIPS is able to achieve better tracking performance with gyroscope drift corrected.

The remainder of the paper is organized as follows: Section 2 introduces preliminaries and related works in INS and UWB FIPS and gyroscope drift correction. The proposed INS and UWB FIPS and gyroscope drift correction approach is described in Section 3. Experimental evaluations as well as discussion regarding the performance of the proposed IPS are presented in Section 4. Section 5 presents the conclusions.

## 2. Preliminaries and Related Works

PDR is commonly used in pedestrian tracking as a self-contained IPS and step-based PDR is able to minimize the influence of accelerometer drift by only identifying peaks in acceleration. In scenarios where a smart device is used as the development platform, the situation where a pedestrian holds the smart device in front of the body is commonly considered [7,8,9]. In this holding position, the device is relatively stable along the axis vertical to the ground during walking. Therefore, a simplification can be made assuming that the vertical axis component of the position is constant. The state update function in step-based PDR, where the state *S* is represented with 3-axis coordinates [*x*, *y*, *z*] in a defined Cartesian coordinate system, is presented in:

(1) $$S_{t}=\left[\begin{array}{c} x_{t}\\ y_{t}\\ z_{c} \end{array}\right]=\left[\begin{array}{c} x_{t-1}\\ y_{t-1}\\ z_{c} \end{array}\right]+L_{t}\cdot \left[\begin{array}{c} cos\theta_{t}\\ sin\theta_{t}\\ 0 \end{array}\right].$$

The symbol *t* (*t* > 0), *L_t_* and *θ* denotes the step index, the step length and orientation of the step, respectively and subscript *c* for *z*-axis coordinates indicates that the location along *z*-axis is a constant. Starting from a known initial position, i.e., *S_0_* = [*x_0_*, *y_0_*, *z_c_*], PDR iteratively updates the location of the pedestrian by estimating the length and orientation of each step.

A PDR approach is developed in our previous work [10] and is employed as the INS subsystem in the FIPS in this paper. The step length is modeled proportional to the square root of step frequency as shown in:

(2) $$L_{t}=c_{s}\cdot \sqrt{f_{s}},$$

with unit in meter, where *f_s_* is the step frequency and *c_s_* is a constant scaling factor. The step orientation is obtained from the yaw component of gyroscope data, which indicates the heading angle of the pedestrian as the *θ* in Equation (1), by finding the average of yaw data within the interval of a detected step. More details can be referred in [10].

Sensor drift over time will deteriorate the performance of PDR-based IPS and information fusion combining wireless technologies is an effective approach to mitigate performance deterioration. UWB technology is increasingly employed in FIPS for enabling accurate distance measurements between UWB nodes. Typically, the INS and UWB subsystems generate location estimation of the pedestrian separately. The two locations are subsequently fused for a final estimation. An unbiased finite impulse response filter is designed in [16] to correct the difference between the INS-measured position and UWB-measured position. By using anti-magnetic ring to eliminate the outliers from the UWB distance measurements, a double-state adaptive KF is proposed in [17] to improve the positioning accuracy during information fusion. The feature of independent position estimation in UWB subsystem, achieved by trilateration using the distances measured between UWB anchor nodes and the pedestrian, inevitably requires that at least 4 UWB anchor nodes are available to provide valid distance measurements for every possible location. In a complex indoor environment where the coverage of a UWB anchor is limited, the total number of required anchors will increase significantly in order to cover the entire indoor space. Alternatively, the distance measurements can be used directly in information fusion using PF [19] and the approach works for arbitrary number of anchor nodes. The FIPS developed in this paper also directly uses distance measurements to ensure that the IPS can be readily extended to cover larger area with optimal cost-effectiveness.

Despite the fact that UWB-enabled nodes are able to measure distances up to 100 m with errors in the order of centimeters in ideal cases, the error may rise up to more than 1.5 m when the direct line-of-sight between UWB nodes is obstructed [19]. The non-line-of-sight (NLOS) condition between UWB nodes is the main source of distance measurement error and existing works have proposed several analytical models, including linear [20], polynomial [21], Gaussian [22] and univariate skew-*t* distributions [23], for error mitigation. Extensive UWB distance measurement campaigns and error profiling experiments are conducted in [14] and a distance measurement error model combining a Gaussian and a gamma distribution is proposed. As formulated in:

(3) $$f\left(\varepsilon \right)=\frac{1}{\sigma \sqrt{2\pi }}\cdot e^{-\frac{{\left(\varepsilon -\mu \right)}^{2}}{{2\sigma }^{2}}}+\lambda \cdot e^{-\lambda \varepsilon }\cdot \frac{{\left(\lambda \varepsilon \right)}^{k-1}}{\Gamma (k)}{+c}_{0},$$

where *ε* denotes the distance error, the first term is a Gaussian distribution with mean and Standard Deviation (SD) represented by *μ* and *σ*, the second term is a gamma distribution defined by parameters *λ* and *k*, and the last constant term *c_0_* is equal to 3% of the Gaussian–gamma combined model’s peak [14].

Apart from the distance measurement error modeling in UWB subsystem, the error modeling of INS subsystem should also be investigated to enhance the performance of the overall FIPS. In step-based PDR, the error of step length estimation can be modeled with a Gaussian distribution [19]. The error of step orientation, on the other hand, is modeled with a Gaussian random walk when the gyroscope data are the angular rotation rate [23] or a Gaussian distribution when heading angle is obtained from gyroscope [24]. An autoregressive and autoregressive moving-average model is built to model the gyroscope random drift with the help of Allan variance [25]. The determined error models for INS errors can be incorporated in FIPS using KF/EKF.

It is observed that the step orientation obtained from gyroscope is stable when the pedestrian is walking in a straight line, i.e., the difference between angle samples along the straight walk is relatively small. Gyroscope drift is accumulated when the pedestrian makes a turn. For instance, considering the case when a pedestrian is walking straight at 0° initially and continues walking straight at 60° after a 60° turn, the gyroscope may report a heading angle at 65° after the turn. The value of drift in this paper is defined as the difference between the ground truth direction and the reported direction by gyroscope. Therefore, in the example, a −5° drift is accumulated during the turn and will continue to affect the tracking performance afterwards. The observation suggests that gyroscope drift correction can be more scrutinized in situations where a turn is made. In [26], two UWB networks are built and installed in two corners (places where a turn is made) of a rectangular path (5 m × 25 m). Each UWB network has a supporting region of a sphere of 4-m radius and tries to correct the gyroscope drift when the pedestrian passes through the UWB network using KF. The main drawback of this approach is that a large number of UWB anchor nodes are required with a total number of 15 nodes (8 nodes for one network and 7 for the other). A turn detection and heading correction approach is proposed in [27] by using pelvic rotation and zero-velocity update. The approach relies on waist mounted IMU sampling pelvic rotation data and thus has limited applicability.

In this paper, a turn detection and gyroscope drift correction approach in an INS and UWB FIPS is proposed. Distance measurements from UWB subsystem are used in the fusion, and the number of required UWB anchor nodes can be significantly reduced. The turn detection and drift correction only rely on the step orientations and estimated locations of the INS subsystem and the fused output. Therefore, the developed approach is applicable to a range of IPSs using different data sources and hardware platforms.

## 3. Proposed Approach

This section consists of four parts. First, a PF-based INS and UWB FIPS is described where only distance measurements from UWB subsystem are used. Second, a turn detection method is detailed in Section 3.2. Third, the turn-based gyroscope drift estimation approach, which calculates the gyroscope drift based on information fusion, is presented. Lastly, the FIPS with gyroscope drift correction incorporating the components in Section 3.1, Section 3.2 and Section 3.3 is proposed.

### 3.1. Distance Measurements Based INS and UWB Fusion

In this study, a PF algorithm is used to fuse the INS and UWB subsystems using only distance measurements from the UWB subsystem. PF is a powerful tool addressing Bayesian state estimation problems characterized by a state update function and an observation function. The distribution of the additive noise can be arbitrary since PF uses a group of particles to estimate the posterior distribution of the system state. In an INS and UWB FIPS, the state update function corrupted by noise can be formulated as in

(4) $$S_{t}=\left[\begin{array}{c} x_{t}\\ y_{t}\\ z_{c} \end{array}\right]=\left[\begin{array}{c} x_{t-1}\\ y_{t-1}\\ z_{c} \end{array}\right]+\left(L_{t}+\psi_{t}\right)\cdot \left[\begin{array}{c} cos\left(\theta_{t}+\delta_{t}\right)\\ sin\left(\theta_{t}+\delta_{t}\right)\\ 0 \end{array}\right],$$

by introducing additive noise associated with the step length and step orientation estimation process in Equation (1), denoted by *ψ* and *δ*, respectively. Each available anchor node makes an observation as the distance between the anchor node and the pedestrian. The observation function is shown in

(5) $$D_{t}^{i}=\left|A^{i}-S_{t}\right|+\varepsilon_{t},$$

measuring the Euclidean distance, $D_{t}^{i}$, between the location of the anchor, *A^i^* = [$x_{a}^{i}$, $y_{a}^{i}$, $z_{a}^{i}$] and *S_t_*. The distance is subject to additive noise, i.e., *ε*, corresponding to the measurement error in the UWB subsystem, and the distribution model of the error shown in Equation (3) is used in this study.

Given that a total number of *I_t_* anchor nodes are available at step index *t*, the PF algorithm iteratively estimates the system state based on $D_{t}^{i}$ distance measurements, {$D_{t}^{i}$, *i* = 1 … *I_t_*}. Particles are sampled from a proposal distribution where the state update function is commonly used [13]. The weight of a particle, *ω*, can then be assigned according to

(6) $$\omega_{t}^{m_{i}}\propto \omega_{t-1}^{m_{i}}\cdot 𝓅(D_{t}^{i}|s_{t}^{m}),$$

as the conditional probability of obtaining a measurement of $D_{t}^{i}$ when the particle state is $s_{t}^{m}$, multiplied by the corresponding particle weight in the previous iteration. With the total number of particles defined as *M*, the superscript of weight, i.e., *m_i_*, represents the *m*th particle corresponding to the *i*th distance measurement.
**Algorithm 1** Inertial navigation system (INS) and ultra-wideband (UWB) fusion algorithm.1. Initialization: 2.           initialize position estimate ${\tilde{S}}_{0}$ and the particle set {$s_{0}^{m}$ = [$x_{0}^{m}$, $y_{0}^{m}$, $z_{0}^{m}$]^T^, (*m* = 1…*M*)} with known position *S_init_* = [*x_0_*, *y_0_*, *z_c_*]^T^ 3.           *K* UWB anchors are installed at {*A^k^* = [$x_{a}^{k}$, $y_{a}^{k}$, $z_{a}^{k}$], *k* = 1…*K*} 4. *Update*: 5.           at step index *t* 6.           obtain step length *L_t_* and orientation *θ_t_* from INS subsystem 7.           *if* no UWB measurement is available, i.e., *I_t_* = 0 8.                    update ${\tilde{S}}_{t-1}$ to ${\tilde{S}}_{t}$ according to Equation (1), i.e., no additive noise 9.                    update particle set {$s_{t-1}^{m}$, (*m* = 1…*M*)} to {$s_{t}^{m}$, (*m* = 1…*M*)} according to Equation (1), i.e., no additive noise 10.           *else* 11.                    obtain $D_{t}^{i}$ UWB distance measurements {$D_{t}^{i}$, *i* = 1…*I_t_*} and corresponding anchor locations {*A^i^*, *i* = 1…*I_t_*}, which are available for the current step (*I_t_* ≤ *K*) 12.                    update particle set {$s_{t-1}^{m}$, (*m* = 1…*M*)} to {$s_{t}^{m}$, (*m* = 1…*M*)} according to Equation (4) 13.                    *for* each $D_{t}^{i}$ with anchor position *A^i^* (*i* = 1…*I_t_*) 14.                             *for* each particle $s_{t}^{m}$ (*m* = 1…*M*) 15.                                      assign particle weight $\omega_{t}^{m_{i}}$ according to Equation (3), (5) and (6) 16.                             *end for* 17.                             normalize {$\omega_{t}^{m_{i}}$, (*m* = 1…*M*)} such that $\overset{M}{\underset{m=1}{\sum }}\omega_{t}^{m_{i}}=1$ 18.                    *end for* 19.                    weight of each particle $\omega_{t}^{m}=\overset{I_{t}}{\underset{i=1}{\sum }}\omega_{t}^{m_{i}}$ 20.                    normalize {$\omega_{t}^{m}$, (*m* = 1…*M*)} such that $\overset{M}{\underset{m=1}{\sum }}\omega_{t}^{m}=1$ 21.                    generate position estimate ${\tilde{S}}_{t}=\overset{M}{\underset{m=1}{\sum }}\omega_{t}^{m}\cdot s_{t}^{m}$ 22.                    Resample {$s_{t}^{m}$, (*m* = 1…*M*)}, using Systematic resampling given {$\omega_{t}^{m}$, (*m* = 1…*M*)} 23.                    go to Update when a new step is detected at index *t*+1 24.           *end if*

The pseudocode of the PF-based fusion algorithm is summarized in Algorithm 1. During initialization, the initial position estimate is set to the known starting position as well as the whole particle set with *M* particles. *K* UWB anchor nodes are installed in the target environment. The iterative processing flow of the algorithm is triggered upon the detection of a step with step length and orientation estimated by the INS subsystem. Since the current position may not be covered by any UWB anchor node, it is always first checked whether UWB measurement is available. If not, the estimated position, as well as the particle set, is updated directly by the INS subsystem according to Equation (1). If a subset of *I_t_* anchor nodes are available, $D_{t}^{i}$ distance measurement(s) will be generated, where *i* = 1...*I_t_* and *I_t_* ≤ *K*. The measurement(s) from UWB subsystem is(are) fused by the PF algorithm. For each available distance measurement, a subset of particle weights is generated correspondingly. Therefore, *I_t_* subset(s) of particle weights will be generated each containing *M* weights. Each subset of the weights is normalized separately first before summing up, in order to avoid the unbalanced weight ranges across different subsets. The final weight of each particle is calculated as the sum of all the subset(s). The normalized final weight set is used both in calculating the position estimation as the weighted sum of the particle set and in resampling the particle set where Systematic Resampling algorithm [28] is employed.

The PF fusion algorithm is flexible as it is able to accommodate arbitrary number of UWB anchor nodes and only fuses available distance measurement(s) at any given step. When no distance measurement is available, the algorithm falls back to PDR and solely rely on the INS subsystem. By employing the fusion algorithm, there is no constraint on the required number of UWB anchor nodes in the developed IPS.

### 3.2. Turn Detection

The turn detection approach proposed in this paper relies on the heading orientation of walking. In a step-detection-based IPS, the system is able to keep track of the step orientation history. Despite that the INS and UWB FIPS described in Section 3.1 does not generate a step orientation in position update directly, the step orientation can be derived from the fused position estimation set, {${\tilde{S}}_{t}$}. The approach detects the walking pattern where the pedestrian keeps a straight walk both before and after making a turn, which is common in indoor environments including office space, laboratories and corridors. The behavior of straight walk is characterized by step-orientation data where the values of the orientation angles are close to each other.

The detailed processing flow of the turn detection approach is as follows: The IPS keeps track of a moving window of step orientations with window size *P*, i.e., the step orientations of the most recent *P* steps represented by {*O_p_*, *p* = 1...*P*}. With a straight walk detection window size represented as *Q*, the first *Q* samples and last *Q* samples of the step orientation window are extracted for straight walk detection (*P* > 2*Q*). The heading angle ranges of the two sample windows, denoted by *R_A1_* and *R_A2_*, as well as the average heading angle, denoted by *A_A1_* and *A_A2_*, are then calculated. Due to the value range of gyroscope data, the ambiguity issue between the lower and upper bound of gyroscope data needs to be addressed [10] when calculating heading angle range and average heading angle. For instance, considering the output heading angle of a gyroscope is (−180°, 180°), the heading angle range between −179° and 178° is 3° instead of the numeric range 357°. Similarly, the average heading angle is 179.5° instead of the numeric average −0.5°. A straight walk is identified when the heading angle range of the sample window is within a defined threshold, *TH_S_*. In this paper, *TH_S_* is set to 5°—based on analysis of the heading orientation data obtained from gyroscope when a pedestrian is walking straight to account for minor fluctuations. A turn is detected when both *R_A1_* and *R_A2_* is less than *TH_S_* and the heading angle difference between *A_A1_* and *A_A2_* is greater than a defined threshold, *TH_T_*. The value of *TH_T_* corresponds to the minimum degree of turn to be detected in ideal case. In practice, the setting of the threshold should also take into consideration possible gyroscope drift and fluctuation. The pseudocode of the turn detection approach is summarized in Algorithm 2. The turn detection algorithm is applied to step orientation samples from both the INS subsystem and fused output. A gyroscope drift estimation is triggered only when a turn is detected for both orientation samples.
**Algorithm 2** Turn detection algorithm  1. Given a window of *P* consecutive step orientations, {*O_p_*, *p* = 1...*P*}  2. Obtain a sub-window of the first *Q* samples, {*O_Fq_*
∈
*O_p_*, *q* = 1...*Q*}  3. Obtain a sub-window of the last *Q* samples, {*O_Lq_* ∈
*O_p_*, *q* = 1...*Q*}  4. Calculate the heading angle range and average heading angle of {*O_Fq_*, *q* = 1...*Q*} as *R_A1_* and *A_A1_*  5. Calculate the heading angle range and average heading angle of {*O_Lq_*, *q* = 1...*Q*} as *R_A2_* and *A_A2_*  6. Calculate the heading angle difference of {*A_A1_*, *A_A2_*} as *R_A_*  7. *if R_A1_* < *TH_S_ and R_A2_* < *TH_S_ and R_A_* > *TH_T_*  8.           *turn detected*  9. *else* 10.           *turn not detected* 11. *end if*

Unlike turn detection algorithms using data from sensors mounted on specific part of the body [27] that are only applicable to systems using the same sensors, the proposed turn detection only relies on moving orientation of the pedestrian, which is an essential information provided by all IPS. Therefore, it can be readily applied in different IPS with varying hardware setup. Moreover, the computation operations involved in the turn detection approach is significantly less complex compared to thresholding the SD of heading orientations.

### 3.3. Gyroscope Drift Estimation

**Figure 1:**
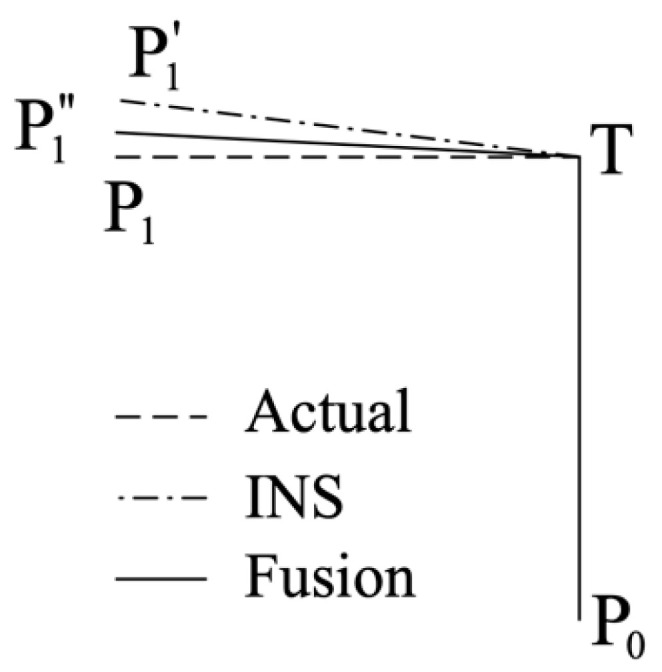
Gyroscope drift estimation.

The gyroscope drift estimation approach aims to find out the accumulated gyroscope drift when a turn is made. The principle of the correction approach illustrated in Figure 1 is based on an ideal 90° turn case and described as follows: It is noted that the proposed approach is able to support arbitrary turning angle above the threshold value *TH_T_*. A person walks along the path from position *P*_0_ to *P*_1_ passing a turning point *T* where a 90° turn is made. Initially, the gyroscope is drift-free. Certain amount of drift is accumulated when making the turn and the traveled path tracked by INS is affected by the drift afterwards, which will lead to the estimated path to be along *T* to *P*_1_’. By applying the INS and UWB fusion algorithm described in Section 3.1, the tracking path after fusion may be along *T* to *P*_1_’’ after the turn. The amount of the accumulated gyroscope drift can be calculated as the difference between ∠*P*_0_*TP*_1_’ and ∠*P*_0_*TP*_1_’’. The exact gyroscope drift can be obtained when the fusion algorithm gives perfect estimation of the position.

**Figure 2:**
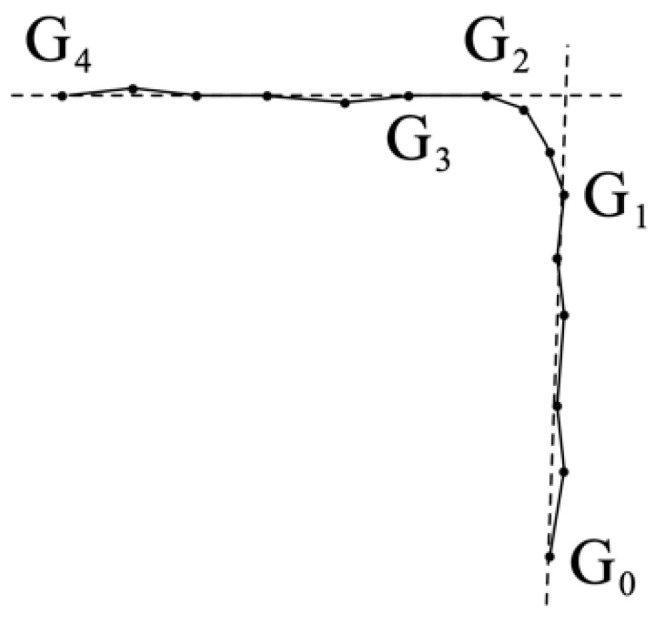
Turning angle estimation.

In practice, the INS and fused path are not ideally straight during a straight walk and the tracking path is generated by accumulating line segments, where each segment corresponds to a step update. A typical case is shown in Figure 2 where a person travels along the path from ***G*_0_** to ***G*_4_**. The tracking path consists of 14 segments that correspond to 14 separately estimated step lengths and orientations. Despite that the person is walking straight before and after the turn, minor fluctuations in step orientations are visible in the generated path.

In order to calculate the gyroscope drift, it is important to estimate the turning angle based on the tracking path. The turning angle estimation approach proposed in this paper combines the turn detection algorithm described in Section 3.2. The turning angle estimation is triggered when a valid turn is detected, indicating that the person is walking straight before and after the turn. Using the same notations introduced in Section 3.2, the turn detection algorithms identifies two sub-windows corresponding to the step orientations during the straight walking behavior. For instance, given *P* = 14, *Q* = 5 and the tracking path is illustrated in Figure 2, a turn is detected with the two sub-windows containing orientation samples for *G*_0_ to *G*_1_ and *G*_3_ to *G*_4_, respectively. Attempts to extend both sub-windows to include more remaining samples are made while ensuring the extended sub-windows correspond to straight walking behavior, i.e., the heading angle range of both sub-windows remains within *TH_S_*. The rationale for extending the straight walking window is that the walking orientation estimated using more step orientation samples will be less sensitive to random noise in gyroscope data. In the example presented in Figure 2, the sub-window representing path *G_0_* to *G_1_* remains unchanged and the other sub-window is extended by one more sample to include *G_2_*. The implementation of the sub-window extension scheme also decouples the generated final sub-windows from the parameter setting of *Q*. For the illustrated example, setting *Q* ≤ 5 will all generate the same sub-windows after extension.

After extending the sub-windows corresponding to straight walking paths, the turning angle estimation approach relies on the starting and ending position of the paths. For each path, a vector is formed pointing to the ending point from the starting point of the path, i.e., $\vec{G_{0}G_{1}}$ and $\vec{G_{2}G_{4}}$ in Figure 2 as indicated by the dotted lines. The estimated turning angle is then calculated as the angle between the two vectors. It is noted that the turning angle estimation approach utilizes the estimated positions only and is also applicable to different types of IPS without any constraint on how the sensors are mounted. Using the starting and ending position of a straight walking path to estimate the orientation also smooths the fluctuations in raw orientation data.

When a gyroscope drift estimation is triggered, the turning angles for INS and the fused position solutions are estimated using the aforementioned estimation approach, represented by ${TA}_{INS}^{r}$ and ${TA}_{fused}^{r}$, respectively. *r* is the index for valid turn occurrences. The estimated gyroscope drift during *r^th^* turn, *dr^r^*, equals to the difference between ${TA}_{fused}^{r}$ and ${TA}_{INS}^{r}$, as shown in:

(7) $${dr}^{r}={TA}_{fused}^{r}-{TA}_{INS}^{r}.$$

It is noted that the sign of the estimated gyroscope drift is subject to adjustment according to the polarity of gyroscope data. In a right-handed convention where counter-clockwise rotation results in positive angular change, the calculated gyroscope drift needs to be negated during a clockwise turn.

Using the presented gyroscope drift estimation algorithm, a delay is observed in estimating the drift when a turn is detected. As shown in Figure 1, the drift may be present in the gyroscope data as soon as the turn is made. A few more steps are required to enable the algorithm to estimate the turning angle for subsequent drift estimation. Despite the delay in gyroscope drift estimation, the overall localization and tracking performance is not influenced by the delay since the UWB distance measurements are incorporated in the FIPS.

### 3.4. Proposed FIPS with Gyroscope Drift Correction

**Figure 3:**
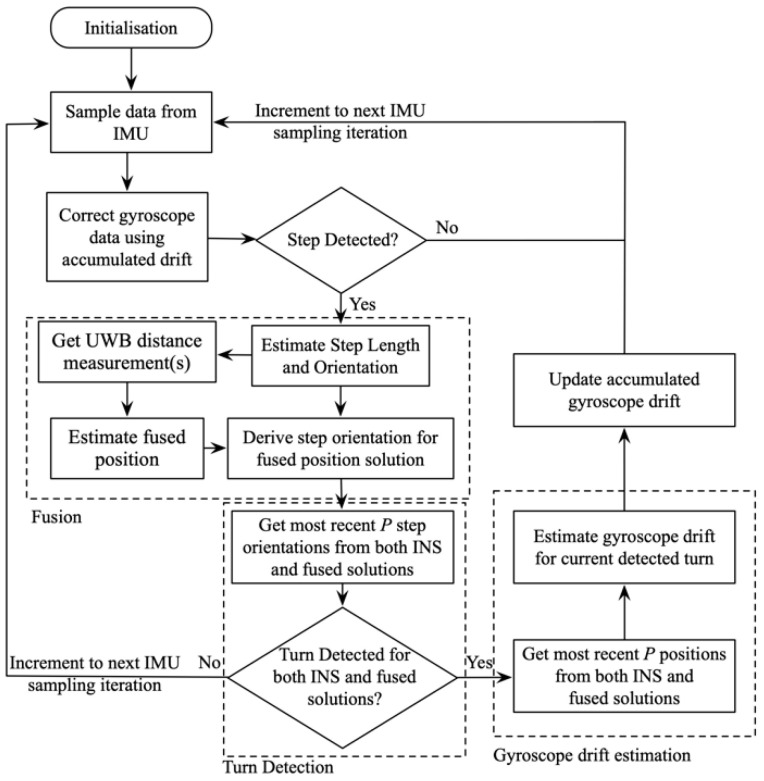
Processing flow of the proposed UWB fusion IPSs (FIPSs).

The processing flow of the proposed FIPS with gyroscope drift correction is illustrated in Figure 3, with components introduced in Section 3.1, Section 3.2 and Section 3.3 grouped by a dashed block. Applying the fusion algorithm presented in Section 3.1, the system keeps track of the most recent *P* samples of step orientations and updated positions for both INS subsystem and the fused solutions. The orientation samples are used for turn detection using the approach described in Section 3.2. When gyroscope drift estimation is triggered, the positions samples are employed to estimate the gyroscope drift for the current turn. Since the gyroscope drift is estimated independently for each turn, the accumulated gyroscope drift, *DR*, equals to the sum of all drift values calculated for all previously calculated drifts as shown in:

(8) $$DR=\sum_{r=1}^{R}{dr}^{r},$$

where *R* denotes the total number of drift estimations, and the initial value of *DR* is zero. The accumulated gyroscope drift is used to correct the sampled gyroscope data by an addition operation.

## 4. Experimental Evaluations

### 4.1. Proposed FIPS with Gyroscope Drift Correction

The proposed INS and UWB FIPS with gyroscope drift correction is evaluated with practical pedestrian tracking experiments. The INS subsystem is implemented by a smartphone application [10] running on an iPhone 7. The smartphone built-in sensors including accelerometer and gyroscope are sampled at 50 Hz. The sampled acceleration data from accelerometer is used for step detection. When a step is detected, the timestamp of the sampled data is recorded as the step timestamp and is used in step length estimation. With the smartphone held in hand in front of the body, the yaw component of gyroscope data measures the angle turned with respect to an initial reference frame where the heading angle is zero. More implementation details of the INS subsystem are presented in [10].

For the UWB subsystem, TREK1000 development kit from DecaWave [15] is used. The development kit consists of multiple EVB1000 UWB nodes configurable to an anchor node or a mobile (tag) node. The channel frequency and data rate of the UWB communication is configured to 3.993 GHz and 110 kbps, respectively to maximize the covered area of UWB nodes [14]. Under this configuration, the UWB anchor nodes report distance measurements between the anchor nodes and the mobile node at 3.57 Hz. Two anchor nodes are used in the experiment. A laptop is connected to a primary anchor to record the distance measurements from both anchors (the primary anchor has access to distance measurements from all anchors) as well as timestamping the distance measurements. Since the update frequency of distance measurements is higher than typical walking frequency, an extra measurement selection process is implemented in the INS and UWB FIPS. When a step is detected, the timestamp of the step is used to select the specific distance measurements, with associated timestamps closest to the step timestamp. As a result, only the selected measurements are used in the fusion process and the remaining ones are discarded. An EVB100 node configured as a mobile node is attached to the back of the smartphone. When carried in hand, the height of the mobile node is at 1.3 m (*z_c_* in Equation (1)). The system time of the smartphone and the laptop is synchronized using Network Time Protocol.

**Figure 4:**
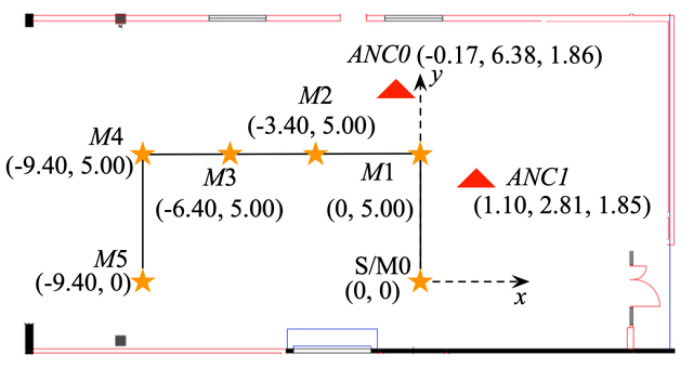
Experiment area layout.

The pedestrian tracking experiment is conducted in Room 332 at the Newmarket campus of the University of Auckland, which is a 10.9 m × 21.0 m laboratory with electrical equipment and rows of computer desks. The layout of the experiment area is illustrated in Figure 4 in a top-view with a defined Cartesian coordinate system. The distances shown in the figure are in meters. The solid line outlines the experiment path with six markers placed on the ground along the path, represented by stars in the figure. The subject starts at position *S*, walks along the path towards *M5* and return to *S*. Considering the path *S* to *M5* to *S* as one round, the experiment path repeated the path for eight rounds, covering a total distance of 310.4 m with 48 turns including 16 left turns, 16 right turns and 16 U-turns, respectively. The markers are used as sample points to calculate position error with the coordinates labeled in Figure 4. When the subject passes the marker, the timestamp is recorded. The timestamp is used as a reference to select a position estimation whose associated timestamp is the closest to the reference. Position error is calculated as the 2D (*x*- and *y*-axis) Euclidean distance between the selected position and the location of the marker. At the same time, heading orientation error is profiled against the ground truth during straight walking. Two anchors are mounted on tripods and installed at the locations denoted by triangles in Figure 4. The height of *ANC0* and *ANC1* is 1.86 m and 1.85 m, respectively. The ground truth coordinates are measured using a Parallax SF11 laser altimeter [29]. The altimeter measures distance in range 0.1 m to 120 m with 1 cm resolution and ±10 cm accuracy.

Regarding parameter settings which are summarized in Table 1, the additive noises for step length and orientation estimation in the state update function, Equation (4), are set to zero mean Gaussian distributions with SD equals to 0.05 and 0.2 [30], respectively. The parameters of the UWB distance measurement error model, including *μ*, *σ*, *λ* and *k* in Equation (3), are kept the same as in [14]. Regarding the setting of *P* and *Q* parameters, it is first, noted that the value of *Q* corresponds to the number of steps required to identify straight walking behavior. In order to cover the straight walking path before and after making a turn, the overall step orientation sample window size *P* must be greater than 2*Q*. The value P-2*Q* corresponds to the number of steps required in making a turn and the two *Q* step orientation sample windows correspond to the path before and after the turn. With a selected P-value, a smaller *Q* value will only ensure the sampled path before and after the turn corresponds to straight walking behavior. If the straight walking behavior is not maintained for the remaining samples (excluding the steps during a turn), the estimated gyroscope drift may be erroneous. It is observed that 3 steps are usually required during a turn. Given the size of the experiment path, *Q* is set to 6 and *P* equals to 15 in this paper. *TH_S_* is set to 5° as described in Section 3.2. Since the minimum turning angle in the experiment is 90°, the value of *TH_T_* is set to 55° by subtracting fluctuation margin (5°) and possible gyroscope drift (maximum value of about 30° [30]) from the minimum turning angle. The number of particles used in the PF fusion algorithm also follows the settings in [14] and equals to 2000.

### 4.2. Results and Discussion

Two independent experiments are conducted, which took 334.1 s and 329.9 s to complete, respectively. The accuracy of the step detection and traveled distance estimation for the INS subsystem is reported in Table 2 with respect to actual steps and traveled distance. For the first experiment, both the step detection rate and traveled distance accuracy are above 93%. The rate drops to 91.15% and 91.89% for step detection and traveled distance, respectively, in the second experiment and is mainly caused by undetected steps, which is more likely to happen when the pedestrian is making a sharp U-turn at either end of the experiment path.

Three different algorithms are applied offline to the collected data from INS and UWB subsystem, which generate three tracking paths to demonstrate the effectiveness of the proposed FIPS with gyroscope drift correction, including: (a) INS, tracking path directly generated by the INS subsystem; (b) Fusion, tracking path generated by applying the INS and UWB fusion algorithm; (c) Fusion-Cor, tracking path generated by the FIPS with gyroscope drift correction.

**Figure 5:**
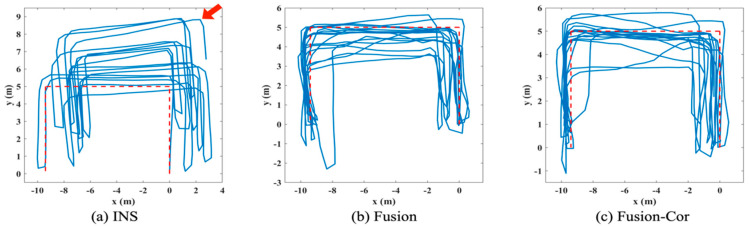
Tracking path for the first experiment.

**Figure 6:**
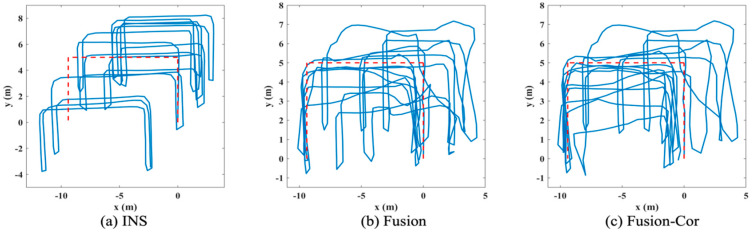
Tracking path for the second experiment.

**Figure 7:**
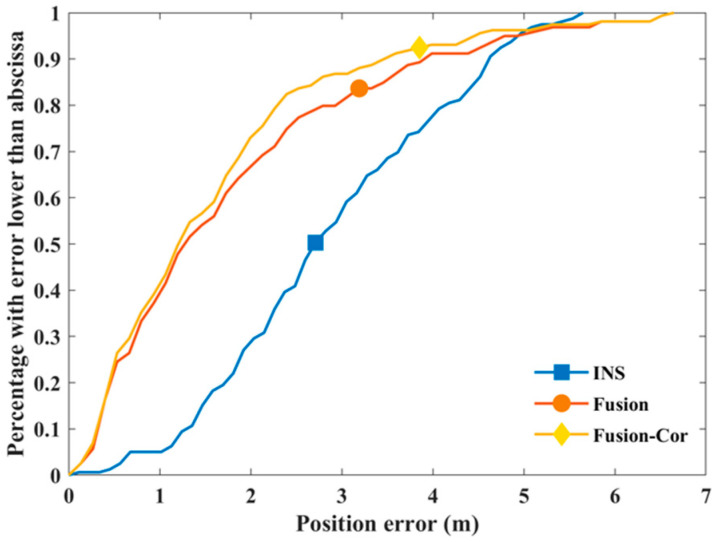
Cumulative distribution function (CDF) of position error.

The tracking paths for the two experiments are presented in Figure 5 and Figure 6, respectively. The quantified mean position error of each path is summarized in Table 3. In Figure 5a, the INS tracking path is clearly affected by gyroscope drift in latter phase of the experiment with path rotated anti-clockwise, which is annotated with a red arrow in figure. The mean position error is 2.48 m. By fusing UWB distance measurements obtained from two anchors, the mean position error is reduced by 60.08% to 0.99 m. By incorporating gyroscope drift correction in fusion, the gyroscope drift estimation is triggered twice during the tracking period. The estimated drift is −4.95° and −2.14°, respectively and the final accumulated gyroscope drift is −7.09°. With drift corrected gyroscope data, the mean position error is further reduced to 0.88 m, achieving a 64.52% reduction compared to the INS result. Regarding the second experiment, no significant gyroscope drift is observed, and the accuracy of the INS solution is mainly affected by undetected steps as shown in Figure 6. The INS subsystem is performing not as good as in the first experiment, which indicates that the state update model is not accurate enough. As a result, the Fusion solution also suffers from larger position errors and only achieves a 22.32% mean position error reduction from 3.27 m to 2.54 m. Since only distance measurements from two UWB anchors are used in the fusion, the UWB subsystem is unable to obtain a position estimation for improved correction performance. This is a tradeoff made to have a more cost-effective solution with fewer number of UWB anchors deployed. Three gyroscope drift estimations are triggered in the second experiment and the calculated drift for each estimation is 0.03°, −0.18° and −1.08°, respectively. The trivial value of drift is verified by the generated tracking path in Figure 6a. Despite the fact that gyroscope drift is not significant, the FIPS with drift correction manages to reduce the mean position error to 2.18 m, achieving a 33.33% reduction compared to INS. The cumulative distribution function (CDF) of position error is further shown in Figure 7 by combining all position error samples in the two experiments. It is observed from figure that the position error of the proposed approach is more concentrated on values less than 2 m when compared to the other two results. This result further strengthens our expectation that information fusion alone is not sufficient and sensor drift correction is needed to achieve a better tracking accuracy.

The tracking performance is compared to existing works that applied UWB and INS fusion and is summarized in Table 4, in terms of mean position error, the total traveled distance of the evaluation experiment, the number of UWB anchors used and path characteristics including the number and type of the turns made. Regarding mean position error, best performance is seen in [16] and [18] where the error is within 0.33 m. However, it is noted that the length of the experiment path is relatively short without repetitive loops and the performance for a longer path is not explored. An experiment path traveled over 1 km is used in [26] by repeating a rectangular path 19 times and the achieved mean position error is 1.05 m. The experiment path used in this paper repeats the U-shaped path 16 times and contained significantly more U-turns. The mean position error for the first experiment outperforms that in [26]. It should be noted that the less accurate tracking performance in the second experiment is caused by undetected steps and it is more likely to happen when the pedestrian is making U-turns, which are not included in the evaluation paths in [16] and [26] and only 2 are included in [18]. Moreover, regarding the number of UWB anchors used, the IPS developed in this paper requires only two anchors and the fusion is based on distance measurements only. It removes the constraint that at least four anchors are required to obtain a position estimation by trilateration and significantly reduces the infrastructure requirement of the developed system.

**Figure 8:**
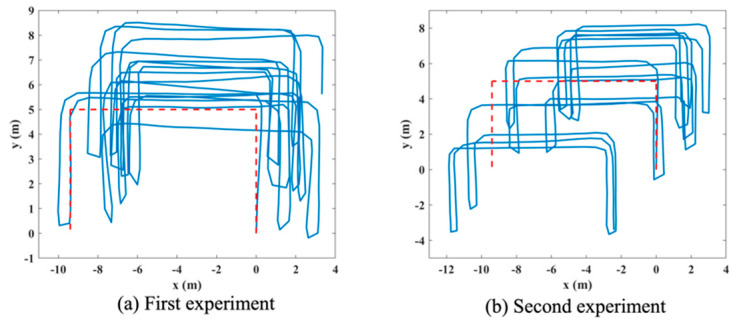
INS-Cor tracking paths.

The above presented tracking path is mainly focused on verifying the positioning accuracy improvement of the proposed approach. The accuracy of the calculated gyroscope drift is not obvious in the generated paths. Therefore, additional tracking paths are generated using step length estimates and the corrected gyroscope data applying INS-based PDR directly, termed as INS-Correction (INS-Cor). The paths are shown in Figure 8a,b for the first and second experiment, respectively. The path for the first experiment (Figure 5a) is affected by gyroscope drift more significantly. Correspondingly, the path in Figure 8a verifies that the drift is accurately corrected. The top right segment of the path follows more closely in the direction of the ground truth path compared to Figure 5a (the segment annotated with arrow). The quantified mean and standard deviation (SD) of orientation error values are summarized in Table 5, achieving 55.58% and 19.87% mean error reduction when compared to INS only PDR for the first and second experiment, respectively.

In the experiments, it is noted that the number of occurrences of the gyroscope drift estimation is significantly smaller than the actual number of turns made by the pedestrian. The reason is that the gyroscope drift estimation process is triggered only when both the INS subsystem and the fused solution satisfy the turn detection conditions described in Section 3.2. This may be a limitation of the proposed approach since the gyroscope drift accumulated during the turns when gyroscope drift estimation is not triggered is not corrected. Using INS subsystem step orientations only to trigger the drift estimation process will lead to more turns detected, as can be seen from the tracking path in Figure 5a and Figure 6a that the path segments before and after each turn are more likely to satisfy the straight walking requirement in the described turn detection algorithm in Section 3.2. However, if the same straight walking behavior is not observed in the fusion system, the reference turning angle derived from the fusion system may not be accurate and will lead to erroneous gyroscope drift correction. The proposed FIPS takes advantage of the accurate turning angle estimation in information fusion when the aforementioned straight walking behavior is observed and corrects the gyroscope drift in INS subsystem to further enhance the positioning and tracking performance.

## 5. Conclusions

In this paper, an INS and UWB FIPS for pedestrian tracking is proposed. The fusion is based on UWB distance measurements only and is able to work with minimum number of UWB anchor nodes, significantly less than other methods proposed in literature. To enhance the INS subsystem affected by gyroscope drift, a gyroscope drift correction approach based on turn detection is incorporated in the fusion process. The proposed approaches use generic information of IPS including heading orientations and position estimations and are applicable to various IPS with different sensor mounting options. Experimental evaluations by practical pedestrian tracking are conducted to demonstrate the effectiveness and accuracy of the gyroscope drift correction approach. With the drift corrected, the fusion system is able to provide more accurate positioning and tracking estimations compared to INS-based PDR and UWB distance measurements-based fusion approach.

## Figures and Tables

**Table 1:** Parameter settings in the proposed indoor positioning system (IPS).

$\psi_{t}(m)$	$\delta_{t}(\mathrm{rad})$	μ(m)	σ(m)	λ	*K*	*P*	*Q*	*TH_S_*	*TH_T_*	*M*
~ℕ(0, 0.05^2^)	~ℕ(0, 0.2^2^)	0.1	0.055	3.5	2	15	6	5°	55°	2000

**Table 2:** Step detection and traveled distance estimation accuracy.

	Experiment 1	Experiment 2
Detected steps/actual steps	474/502	453/497
Percentage	94.42%	91.15%
Estimated distance/Actual distance (m)	290.45/310.4	285.22/310.4
Percentage	93.57%	91.89%

**Table 3:** Mean position error.

	INS	Fusion	Fusion-Cor
Experiment 1	2.48 m	0.99 m	0.88 m
Experiment 2	3.27 m	2.54 m	2.18 m

**Table 4:** Tracking performance comparison.

	[16]	[18]	[26]	Proposed
Mean position error (m)	0.30	0.33	1.05	0.81/2.18
Total traveled distance (m)	~30	100	1140	310.4
Number of UWB anchors	5	4	15	2
No. of turns made	3	31	78	48
No. of left turns	3	14	0	16
No. of right turns	0	15	78	16
No. of U-turns	0	2	0	16

**Table 5:** Orientation error.

	INS	INS	INS-Cor
Experiment 1	Mean	3.94°	1.75°
	SD	2.39°	1.29°
Experiment 2	Mean	1.56°	1.25°
	SD	1.00°	0.85°

## References

[1] Lin C.-C., Chiu M.-J., Hsiao C.-C., Lee R.-G., Tsai Y.-S. (2006). Wireless health care service system for elderly with dementia. IEEE Trans. Inf. Technol. Biomed..

[2] Barberis C., Andrea B., Giovanni M., Paolo M. (2014). Experiencing Indoor Navigation on Mobile Devices. It Prof..

[3] Paterna V.C., Auge A.C., Aspas J.P., Bullones M.A.P. (2017). ABluetooth Low Energy Indoor Positioning System with Channel Diversity, Weighted Trilateration and Kalman Filtering. Sensors.

[4] Yang C., Shao H. (2015). WiFi-based indoor positioning. IEEE Commun. Mag..

[5] Suski W., Banerjee S., Hoover A. (2013). Using a map of measurement noise to improve UWB indoor position tracking. IEEE Trans. Instrum. Meas..

[6] Ruiz A.R.J., Granja F.S., Honorato J.C.P., Rosas J.I.G. (2012). Accurate Pedestrian Indoor Navigation by Tightly Coupling Foot-Mounted IMU and RFID Measurements. IEEE Trans. Instrum. Meas..

[7] Tian Q., Salcic Z., Wang K.I.-K., Pan Y. An enhanced pedestrian dead reckoning approach for pedestrian tracking using smartphones. Proceedings of the IEEE Tenth International Conference on Intelligent Sensors, Sensor Networks and Information Processing (ISSNIP).

[8] Li Y., Ning F. (2018). Low-Cost Indoor Positioning Application Based on Map Assistance and Mobile Phone Sensors. Sensors.

[9] Tian Z., Zhang Y., Zhou M., Liu Y. (2014). Pedestrian dead reckoning for MARG navigation using a smartphone. EURASIP J. Adv. Signal Process..

[10] Tian Q., Salcic Z., Wang K.I.-K., Pan Y. (2016). A multi-mode dead reckoning system for pedestrian tracking using smartphones. IEEE Sens. J..

[11] Akeila E., Salcic Z., Swain A. (2014). Reducing low-cost INS error accumulation in distance estimation using self-resetting. IEEE Trans. Instrum. Meas..

[12] ADIS 16490 Data Sheet, Analog Devices. https://www.analog.com/media/en/technical-documentation/data-sheets/adis16490.pdf.

[13] Canedo-Rodríguez A., Alvarez-Santos V., Regueiro C.V., Iglesias R., Barro S., Presedo J. (2016). Particle filter robot localisation through robust fusion of laser, WiFi, compass and a network of external cameras. Inf. Fusion.

[14] Ruiz A.R.J., Granja F.S. (2017). Comparing Ubisense, BeSpoon, and DecaWave UWB Location Systems: Indoor Performance Analysis. IEEE Trans. Instrum. Meas..

[15] TREK1000 User Manual, DecaWave. https://www.decawave.com/wp-content/uploads/2018/09/trek1000_user_manual.pdf.

[16] Xu Y., Ahn C.K., Shmaliy Y.S., Chen X., Li Y. (2018). Adaptive robust INS/UWB-integrated human tracking using UFIR filter bank. Measurement.

[17] Fan Q., Sun B., Sun Y., Zhuang X. (2017). Performance enhancement of MEMS-based INS/UWB integration for indoor navigation applications. IEEE Sens. J..

[18] Youssef J., Denis B., Godin C., Lesecq S. Loosely-coupled IR-UWB handset and ankle-mounted inertial unit for indoor navigation. Proceedings of the IEEE International Conference on Ultra-Wideband (ICUWB).

[19] Tian Q., Wang K.I.-K., Salcic Z. (2018). Human body shadowing effect on UWB-based ranging system for pedestrian tracking. IEEE Trans. Instrum. Meas..

[20] Djaja-Josko V., Kolakowski M. A new map based method for NLOS mitigation in the UWB indoor localization system. Proceedings of the 25th Telecommunication Forum (TELFOR).

[21] Ferreira A.G., Fernandes D., Catarino A.P., Monteiro J.L. (2017). Performance Analysis of ToA-Based Positioning Algorithm for Static and Dynamic Target in Low Ranging Measurements. Sensors.

[22] Garcia E., Poudereux P., Hernandez A., Urena J., Gualda D. A robust UWB indoor positioning system for highly complex environments. Proceedings of the IEEE International Conference on Industrial Technology (ICIT).

[23] Nurminen H., Ardeshiri T., Piche R., Gustafsson F. A NLOS-robust TOA positioning filter based on a skew-t measurement noise model. Proceedings of the International Conference on Indoor Positioning and Indoor Navigation (IPIN).

[24] Moder T., Hafner P., Wisiol K., Wieser M. 3D indoor positioning with pedestrian dead reckoning and activity recognition based on Bayes filtering. Proceedings of the 2014 International Conference on Indoor Positioning and Indoor Navigation (IPIN).

[25] Georgy J., Noureldin A., Korenberg M.J., Bayoumi M.M. (2010). Modeling the stochastic drift of a MEMS-based gyroscope in gyro/odometer/GPS integrated navigation. IEEE Trans. Intell. Transp. Syst..

[26] Hartmann F., Rifat D., Stork W. Hybrid indoor pedestrian navigation combing an INS and a spatial non-uniform UWB-network. Proceedings of the 19th International Conference on Information Fusion.

[27] Muhammad M.N., Salcic Z., Wang K.I.-K. (2018). Detecting turns and correcting headings using low-cost INS. J. Navig..

[28] Carpenter J., Clifford P., Fearnhead P. (1999). Improved particle filter for nonlinear problems. IEE Proc.-Radar Sonar Navig..

[29] Parallax SF11 Laser Altimeter Product Manual. https://www.parallax.com/sites/default/files/downloads/28054-SF11-Laser-Altimeter-Manual-Rev-1.pdf.

[30] Tian Q., Salcic Z., Wang K.I.-K., Pan Y. (2015). A Hybrid Indoor Localization and Navigation System with Map Matching for Pedestrians Using Smartphones. Sensors.

